# ANCA-negative EGPA: only eosinophils without vasculitis? Insights from anti-T2 biologics

**DOI:** 10.3389/fimmu.2023.1325299

**Published:** 2023-11-27

**Authors:** Mario Andrea Piga, Paolo Fraticelli, Leonardo Antonicelli, Maria Stella Garritani, Giulia Ghirelli, Matteo Martini, Angelica Di Vincenzo, Maria Giovanna Danieli, Gianluca Moroncini, Maria Beatrice Bilò

**Affiliations:** ^1^ National PhD Course in Precision Medicine in Rare Diseases, University of Palermo, Palermo, Italy; ^2^ Clinica Medica, Internal Medicine Department, Marche University Hospital, Ancona, Italy; ^3^ Allergy Unity, Internal Medicine Department, Marche University Hospital, Ancona, Italy; ^4^ Internal Medicine Residency Programme, Department of Clinical and Molecular Sciences, Marche Polytechnic University, Ancona, Italy; ^5^ Department of Clinical and Molecular Sciences, Marche Polytechnic University, Ancona, Italy; ^6^ Allergy and Clinical Immunology Residency Programme, Department of Clinical and Molecular Sciences, Marche Polytechnic University, Ancona, Italy

**Keywords:** ANCA, biologics, EGPA, eosinophils, vasculitis

## Abstract

The pathogenic role of p-ANCA in eosinophilic granulomatosis with polyangiitis (EGPA) is a long-standing matter of debate. In this work, we report our real-life experience with EGPA patients, treated with biologics targeting type 2 (T2)-eosinophilic inflammation (Mepolizumab, Benralizumab, Dupilumab). Interestingly, we observed EGPA extrarespiratory relapses only in p-ANCA-positive patients (2/5 cutaneous vasculitis, 3/5 constitutional symptoms), with new rise of p-ANCA and normal eosinophil blood count. Notably, revising our cohort with the new ACR 2022 criteria, these five patients were the only ones to satisfy the entry criterion of vasculitis’ defined diagnosis at disease onset. These observations may suggest that biologics, selectively turning off T2 inflammation, may have unmasked p-ANCA exclusive role in the pathogenesis of vasculitis in EGPA. Therefore, we raise the question whether EGPA vasculitis exists only in p-ANCA-positive patients, and whether p-ANCA-negative disease is “only eosinophils without vasculitis”.

## Introduction

Eosinophilic granulomatosis with polyangiitis (EGPA) is an immune-mediated systemic disease, characterized by hypereosinophilia-related damage and necrotizing vasculitis of medium/small vessels (1, 2). Hallmarks are eosinophilic asthma, chronic rhinosinusitis (CRS), especially with nasal polyps (CRSwNP), and peripheral neuropathy. Perinuclear anti-neutrophil cytoplasm antibodies (p-ANCA) are observed only in ~30-35% of cases, and their pathogenetic role is a long-standing matter of debate, while the role of eosinophils is well established. Therapies include corticosteroids, immunosuppressants and Mepolizumab (3, 4), an anti-IL-5 drug directed against type 2 (T2)-eosinophilic inflammation previously approved for the treatment of severe eosinophilic asthma and recently approved also for severe CRSwNP, hypereosinophilic syndrome (HES) and EGPA. Other therapies used in T2 respiratory diseases, such as Benralizumab (anti-IL-5-R, approved for severe eosinophilic asthma) and Dupilumab (anti-IL4/IL-13Rα, approved for severe T2-eosinophilic asthma and CRSwNP), showed efficacy also in the context of EGPA (5, 6). In this work, we discuss about p-ANCA status, reporting our experience with 13 EGPA patients treated with biologics targeting T2-eosinophilic inflammation from 2/2018 to 1/2023 at Internal Medicine Department, Marche University Hospital, Ancona, Italy. Informed consent was obtained for using patients’ data.

## Patients’ baseline characteristics

All patients were classifiable as EGPA according to the old American College of Rheumatology (ACR) 1990 criteria (7). Diagnosis was based on clinical symptoms and radiological, laboratory and histopathological findings. All patients had asthma, CRS, hypereosinophilia and non-fixed pulmonary infiltrates. 10/13 patients had extra-respiratory manifestations. Organ biopsies were performed in 8/13 patients, all showing extravascular eosinophils, with evidence of necrotizing leukocytoclastic vasculitis in 4/8. 5/13 patients were p-ANCA-positive. Patients’ details are reported in **Table 1**.

**Table 1:** Baseline characteristics.

# Patient (sex,age^†^)	EGPA manifestations (time of onset)	Date of EGPA diagnosis	p-ANCA (time of first positivity)	Organ biopsy (date)	Pre-biologic therapy^‡^ (start-stop)
#1 (M, 54)	Asthma (2010), CRSwNP (2012), myopericarditis and constitutional symptoms (9/2013)	9/2013	Neg.	No	AZA→MMF→CSA→MMF (9/2013-2/2018)
#2 (M, 52)	Asthma, CRSwNP (2007), purpura, peripheral neuropathy and constitutional symptoms (3/2008) TIA^§^ (3/2008, 2011, 2018, 2019, 2020)	3/2008	Pos. (3/2008) APS§ triple positivity (11/2021)	Skin (3/2008): necrotizing leucocytoclastic vasculitis with extravascular eosinophils	CSA (7/2008-11/2009) AZA (11/2009-6/2019) Warfarin, LDA^§^ (11/2021-ongoing)
#3 (M, 57)	Asthma, CRSwNP (1985)	2004	Neg.	Lung (2017): extravascular eosinophils	
#4 (F, 62)	Asthma, CRSwNP (1990), periorbital oedema (2017), arthritis, purpura, peripheral neuropathy and constitutional symptoms (9/2019)	9/2019	Pos. (9/2019) RF+ (9/2019)	Lung (1990,2000, 9/2019): extravascular eosinophils Skin (9/2019): necrotizing leucocytoclastic vasculitis with extravascular eosinophils	RTX (11/2019-1/2020) AZA (7-10/2020)
#5 (F, 55)	Subglottic stenosis (2/1995), asthma, CRSwNP (2004), urticaria (2006)	2/1995 Probable; 12/2004 Defined	Pos. (12/2004)	Subglottic tissue (2/1995): extravascular eosinophils forming granulomas and necrotizing leucocytoclastic vasculitis	CYC oral (3-7/1995) AZA (4/1995-5/1997); MMF (3/2015-2/2018)
#6 (F, 62)	Asthma, CRSwNP (1998), urticaria (1999)	11/2013	Neg.	Nasal polyp (5/2014): extravascular eosinophils	AZA (11/2013-5/2014)
#7 (M, 68)	Asthma, CRSwNP (2012)	3/2012	Neg.	No	AZA→CSA→AZA→ CSA→MMF (12/2012-11/2020)
#8 (M, 65)	Asthma, CRS (1966), constitutional symptoms (7/2009)	7/2009	Neg.	No	CSA→AZA→Imatinib →MMF →MTX (7/2009-9/2015)
#9 (F, 51)	Asthma, CRSwNP (2006) Peripheral neuropathy (11/2018)	10/2006	Neg.	Lung (10/2006): extravascular eosinophils	
#10 (F, 53)	Asthma, CRSwNP (1997), urticaria (1998)	1997	Neg.	Lung (1997): extravascular eosinophils	
#11 (F, 68)	CRSwNP (1984), Asthma (1996)	7/2010	Neg.	No	AZA (9-11/2018)
#12 (F, 57)	Asthma, CRSwNP (2014), arthritis, peripheral neuropathy, purpura and constitutional symptoms (10/2018)	10/2018	Pos. (10/2018) RF+ (10/2018)	Skin (10/2018): leucocytoclastic necrotizing vasculitis with extravascular eosinophils	PEX (10/2018) CYC iv (10/2018-3/2019) AZA (4/2019-1/2020) RTX (1/2020)
#13 (F, 74)	Asthma, CRS (2015), periorbital oedema, CNS vasculitis, cardiac arrhytmia (AF), myocarditis and constitutional symptoms (1/2016)	1/2016	Pos. (5/2015)	No, but brain MRI showing CNS vasculitis (1/2016)	RTX (1-2/2016) AZA→MMF(3-10/2016)

^†^Age (years) at last follow-up (1/2023).

^‡^From time of EGPA diagnosis, all patients were treated with corticosteroids.

§In patient #2, diagnosis of APS was made when he was already under Dupilumab treatment.

The symbol "→" represents the switch from one therapy to another.

AF, atrial fibrillation; APS, antiphospholipid syndrome; AZA, azathioprine; CNS, central nervous system; CRS, chronic rhinosinusitis; CRSwNP, chronic rhinosinusitis with nasal polyps; CSA, cyclosporine A; CYC, cyclophosphamide; EGPA, eosinophilic granulomatosis with polyangiitis; LDA, low-dose aspirin; MMF, mycophenolate mofetil; MRI, magnetic resonance imaging; MTX, methotrexate; p-ANCA, perinuclear anti-neutrophil cytoplasm antibodies; PEX, plasma exchange; RF, rheumatoid factor; RTX, rituximab; TIA, transient ischemic attack.

After EGPA diagnosis, all patients were treated with high-dose corticosteroids, and 10/13 with immunosuppressants (**Table 1**). Complete remission of all clinical manifestations was achieved, with normalization of eosinophils, C-reactive protein (CRP), erythrocyte sedimentation rate (ESR), and p-ANCA negativization in positive patients.

>However, despite concurrent immunosuppressants’ use, every attempt to reduce/discontinuate steroids resulted in new eosinophils’ increase associated with CRS-asthma relapses (steroid-dependent patients).

In 2018 and 2019, respectively, Mepolizumab and Benralizumab were approved for severe eosinophilic asthma. Since all our patients were affected by this condition, they started Mepolizumab (100 mg/month) or Benralizumab (30 mg/2 months) even if, at that time, these biologics were not approved for EGPA. For this reason, no approval from research ethics board (REB) was needed.

At Mepolizumab/Benralizumab initiation asthma and CRS were the only active clinical manifestations of EGPA, with Birmingham Vasculitis Activity Score (BVAS) = 2. All patients had normal CRP and ESR, and p-ANCA were negative in previously positive patients. Baseline use of oral corticosteroids (OCS) is reported in **Table 2**. In 12/13 patients, immunosuppressants have been discontinued because of lack of efficacy on respiratory symptoms, remission of extra-respiratory manifestations, side effects.

Table 2Response to biological therapies.

**Table:** 

MEPOLIZUMAB 100 mg/month/BENRALIZUMAB 30 mg/2 months					
# Patient	Pre-biologic OCS	Biologic (start-stop)	Post-biologic OCS^†^	Extrarespiratory relapse	Management of relapse
#1	Chronic use 10 mg/day^‡^	Mepolizumab (2/2018-ongoing)	Stop 2/2020	No	
#2	Chronic use 10 mg/day^‡^ *1 ENT surgery*	Benralizumab (11/2019-10/2020)	2,5 mg/day, but 4 courses at higher (medium) doses	No	
#3	Chronic use 6,5 mg/day^‡^ *4 ENT surgeries*	Benralizumab (11/2018-9/2020)	3,75 mg/day	No	
#4	Chronic use 2,5 mg/day *1 ENT surgery*	Benralizumab (11/2020-ongoing)	Stop chronic use 2/2021, but 2 courses at medium doses	Arthromyalgias, from 11/2021, oscillating symptoms	Wait and see
#5	Chronic use 5 mg/day^‡^	1) Mepolizumab (2/2018) → 2) Benralizumab (7/2020-2/2021)	1) stop chronic use (8/2019), but 6 courses at medium dose; ENT surgery (1st, 7/2018) 2) 1 course at medium doses	Arthromyalgias, from 11/2018, oscillating symptoms,	Wait and see
#6	Chronic use 5 mg/day^‡^ 2 *ENT surgeries*	Mepolizumab (2/2018-ongoing)	Stop chronic use (2/2019), but 5 courses at medium doses; ENT surgery (3rd, 9/2020)	No	
#7	Chronic use 5 mg/day^‡^	Mepolizumab (10/2018-ongoing)	Stop chronic use (6/2019) but 5 courses at medium doses	No	
#8	Chronic use 5 mg/day^‡^	Mepolizumab (3/2019-ongoing)	Stop (10/2019)	No	
#9	Frequent courses at medium doses (4/year) *1 ENT surgery*	Benralizumab (7/2021-ongoing)	No more courses	No	
#10	Chronic use 12,45 mg/day *4 ENT surgeries*	Benralizumab (11/2021-ongoing)	4,68 mg/day	No	
#11	Several courses at medium doses (2/months) *3 ENT surgeries*	Benralizumab (12/2021-ongoing)	2 courses at medium dose	No	
#12	1) Chronic use 5 mg/day^‡^ 2) Chronic use 12,5 mg/day^‡^	Benralizumab 1) 9/2020-5/2021 2) 7/2022-ongoing	1) stop 3/2021; 2) 12,5 mg/day, with 1 course at higher doses	Arthromyalgias, purpura 4/2021 Benralizumab stop 5/2021	Medium-dose steroids; AZA 8-9/2021; RTX 7/2022
#13	1) Chronic use 5 mg/day^‡^ 2) Chronic use 3,75 mg/day^‡^	Benralizumab 1) 4/2021, single administration 2) 11/2022-ongoing	1) not valuable 2) 2,50 mg/day	Arthromyalgias, fever, livedo 4/2021	High-dose steroids; RTX 5/2021

**Table:** 

DUPILUMAB 300 mg/2 weeks					
#Patient	Pre-biologic OCS^§^	Dupilumab (start-stop)	Post-biologic OCS^†^	Extrarespiratory relapse	Management of relapse
#2	1) 2,5 mg 2) 8 mg	1) 11/2020-3/2021 2) 8/2021-ongoing	1) stop (2/2021) 2) stop (1/2023)	Arthromyalgias -3/2021; -11/2021; -6/2022 +fever Dupilumab stop 3/2021	Medium-dose steroids 3/2021, 11/2021, 6/2022; AZA 11/2021-5/2022; RTX 7/2022 Benralizumab 3-5/2021
#3	3,75 mg	11/2020-ongoing	Stop (1/2021)	No	
#5	1 course at medium doses	5/2021-ongoing	No more courses	arthromyalgias started under Mepolizumab, oscillating symptoms	Wait and see

^†^At last follow-up (1/2023) or at drug stop. ^‡^With frequent courses at higher doses. ^§^During the previous biological treatment. The symbol "→" represents the switch from one therapy to another.

AZA, azathioprine; ENT, ear, nose and throat; OCS, oral corticosteroids, expressed in prednisone dose equivalent; RTX, rituximab.

## Response to anti-T2 biologics

Both Mepolizumab and Benralizumab normalized eosinophils in all patients. A significative improvement in asthma control was observed, as documented by asthma control test and lung function tests (data not reported), and by OCS reduction/discontinuation (reported in **Table 2**).

For incomplete CRSwNP response, negatively impacting on asthma, three patients were switched to Dupilumab (300 mg/2 weeks) with complete control of both upper and lower respiratory symptoms and OCS withdrawal (**Table 2**). Even for Dupilumab treatment, no approval from REB was needed, since patients were affected by severe T2 asthma-CRSwNP (approved indications for Dupilumab prescription). Dupilumab-induced hypereosinophilia was observed, but without related clinical symptoms and not exceeding the described alert cut-off of 3000/mmc (8), except for patient #2 (see below).

During treatment with anti-T2 biologics, we observed EGPA extrarespiratory relapses only in the 5 formerly p-ANCA-positive patients, with new rise of p-ANCA, CRP and ESR.

At the time of relapse, patient #2 was under Dupilumab treatment, patients #4, #12 and #13 were under Benralizumab treatment, and patient #5 was under Mepolizumab treatment. Patients #4 and #5 experienced arthromyalgias under Benralizumab and Mepolizumab treatment, respectively, with eosinophils within the normal range. Since the symptoms were mild, episodic, and self-limiting, and values of p-ANCA, ESR and CRP were low, a “wait-and-see” strategy was adopted. Patient 4 maintained Benralizumab therapy, while patient 5, for incomplete CRSwNP response negatively impacting on asthma, was switched from Mepolizumab to Benralizumab and finally to Dupilumab. Over time, in both patients, symptoms remained stable (mild, episodic and self-limiting), while p-ANCA, ESR, CRP mantained at low levels, often with spontaneous normalization at subsequent checks. Patients #12 and #13 relapsed under Benralizumab treatment, with eosinophils within the normal range. Patient #12 experienced arthromyalgias and purpura of the lower limbs, while patient #13 experienced fever, arthromyalgias, and livedo racemose of the arms. Both patients were then treated with steroids and Rituximab (RTX), with clinical remission and p-ANCA persistent negativization, even after steroids withdrawal.

Patient #2 experienced three extrarespiratory relapses under Dupilumab treatment. At first relapse (arthromyalgias), after OCS withdrawal, both p-ANCA and eosinophils were elevated; particularly, Dupilumab-induced hypereosinophilia was severe (5000/mmc). Dupilumab was prudentially stopped and the patient was treated with steroids and resumption of Benralizumab. However, at steroid reduction, Benralizumab again proved not to be effective on CRSwNP, so a new attempt with Dupilumab was made, concurrently using medium-dose OCS (prednisone 10 mg/day) to cover a new possible severe hypereosinophilia. After two months, gradual OCS tapering was started, strictly checking the blood count. In this context, two subsequent relapses (arthromyalgias and fever) occurred. During these relapses, eosinophils were stable at a value of 500-1000 cells/mmc, under low-dose OCS (prednisone 5 mg/day), while p-ANCA increased again. The second relapse was treated with temporary re-increase of OCS (10 mg/day) and azathioprine. For the third relapse, Rituximab was administered, with clinical remission and p-ANCA persistent negativization, allowing steroids discontinuation. After OCS withdrawal, eosinophils rose to 1500-2000/mmc, but the patient maintained asymptomatic. Relapses’ managements are reported in **Table 2**.

## Discussion

In patients undergoing Mepolizumab/Benralizumab, eosinophils were within the normal range during extrarespiratory relapses. Therefore, we hypothesized that extrarespiratory relapses might be due to the raised p-ANCA. In patient #2, undergoing Dupilumab, both p-ANCA and eosinophils were elevated during the first relapse, making difficult to establish symptoms’ cause (p-ANCA, hypereosinophilia, or both); particularly, Dupilumab-induced hypereosinophilia was severe (5000/mmc). However, during subsequent relapses, eosinophils were stable at a value of 500-1000/mmc, under low-dose OCS, while p-ANCA increased again. Furthermore, p-ANCA negativization following Rituximab coincided with clinical remission, although eosinophils rose to 1500-2000/mmc after OCS discontinuation. Therefore, also in patient #2, we finally hypothesized that extrarespiratory relapses might be due to the raised p-ANCA. Nevertheless, Dupilumab-induced hypereosinophilia could have played a concurrent role in first relapse, so that data relating this patient should be evaluated with great caution.

Based on the above, in our p-ANCA-positive patients, EGPA seemed to have two distinct pathogeneses, sensitive to different therapies: T2-eosinophilic inflammation and p-ANCA-mediated inflammation, responsive to anti-T2 biologics and immunosuppressants, respectively.

This concept has been reported in literature: while T2-eosinophilic inflammation would be responsible for asthma, CRS and eosinophilic organ damage (e.g. myocarditis, gastroenteritis), p-ANCA would underlie vasculitic manifestations (1, 2). However, the verbatim application of this strict dualism cannot always be translated into routine clinical practice (1), and the pathogenic role of p-ANCA is a long-standing matter of debate. An International Consensus reported their poor sensitivity/specificity in differentiating “vasculitic” from “eosinophilic” EGPA (9), stating that p-ANCA cannot guide treatment decision (9).

Nevertheless, in our experience, biologics, selectively turning off T2-eosinophilic inflammation and allowing OCS reduction/withdrawal, may have unmasked p-ANCA exclusive role in the pathogensis of vasculitis in EGPA. Indeed, even if extrarespiratory relapses were mainly represented by aspecific inflammatory constitutional symptoms, 2/5 patients relapsed with frank cutaneous vasculitis (purpura, livedo racemosa). Moreover, the 5 relapsing p-ANCA positive patients were the only ones to have a defined diagnosis of vasculitis at disease onset (4 bioptic, 1 radiologic), so being the only ones to satisfy the entry criterion of the new ACR 2022 criteria (10).

These observations may raise the question whether EGPA vasculitis exists only in p-ANCA-positive patients, and whether p-ANCA-negative disease is only T2-eosinophilic inflammation without vasculitis.

Assuming this, our p-ANCA-negative patients could be considered as having “only” eosinophilic asthma+CRS and/or hypereosinophilic syndrome (HES). The likely bias in their EGPA misdiagnosis would lie in the old ACR 1990 criteria (7), not considering vasculitis’ defined diagnosis.

In literature, however, evidence of organ vasculitis was reported (albeit rarely) also in p-ANCA negative patients. For these cases, some possible explanations could be hypothesized:

1) confounding manifestations, like neuropathy, that are strongly suggestive of vasculitis but can also be exclusively provoked by T2-eosinophilic inflammation (11–13); unfortunately, neuropathy-involved nerves are not always susceptible to diagnostic biopsy in routinary clinical practice;
2) vasculitis not ANCA-associated (drugs, infections, cryoglobulins) that may occur in patients with eosinophilic asthma and/or HES, leading to a clinical picture misdiagnosed as EGPA;
3) vasculitis-like histopathology of involved organs, due to eosinophil-derived vascular damage and thrombosis, but not properly to an autoimmune vasculitis;
4) false negativity of p-ANCA when patients are already undergoing steroid therapy.

A great limit of our hypotheses is that they derive from observations in a small cohort of EGPA patients. However, similar cases have been recently reported, describing the new onset of p-ANCA vasculitis in patients undergoing anti-T2 biologics for severe asthma. In these patients, despite remission of T2-eosinophilic inflammation, development of vasculitis was observed, accompanied by the new finding of p-ANCA positivity (14–18).

## Conclusion

Biologics, selectively turning off T2-eosinophilic inflammation and allowing OCS reduction/withdrawal, may have unmasked p-ANCA exclusive role in the pathogenesis of vasculitis in EGPA. If confirmed by larger studies, these observations may have important therapeutic implications, possibly leading to a pathogenesis-based therapy of EGPA.

## Data availability statement

The original contributions presented in the study are included in the article/supplementary material. Further inquiries can be directed to the corresponding author.

## Ethics statement

Ethical approval was not required for the study involving humans in accordance with the local legislation and institutional requirements. Written informed consent to participate in this study was not required from the participants or the participants’ legal guardians/next of kin in accordance with the national legislation and the institutional requirements. Written informed consent was obtained from the individual(s) for the publication of any potentially identifiable images or data included in this article.

## Author contributions

MP: Conceptualization, Data curation, Writing – original draft, Writing – review & editing. PF: Conceptualization, Data curation, Writing – original draft, Writing – review & editing. LA: Conceptualization, Data curation, Writing – original draft, Writing – review & editing. MG: Data curation, Writing – review & editing. GG: Data curation, Writing – review & editing. MM: Data curation, Writing – review & editing. AV: Data curation, Writing – review & editing. MD: Data curation, Writing – review & editing. GM: Conceptualization, Data curation, Writing – original draft, Writing – review & editing. MB: Conceptualization, Data curation, Writing – original draft, Writing – review & editing.
